# From the archives: BETL, gamma, alpha—an unexpected transporter mediating sucrose transfer in maize, innovation by whole-genome duplication, and cooperative DNA binding by Opaque2

**DOI:** 10.1093/plcell/koad220

**Published:** 2023-08-18

**Authors:** Mariana A S Artur

**Affiliations:** Assistant Features Editor, The Plant Cell, American Society of Plant Biologists; Laboratory of Physiology, Wageningen Seed Science Centre, Wageningen University, Wageningen 6708PB, the Netherlands

## November 2022: An unexpected transporter mediating sucrose transfer in maize

**Figure 1.**
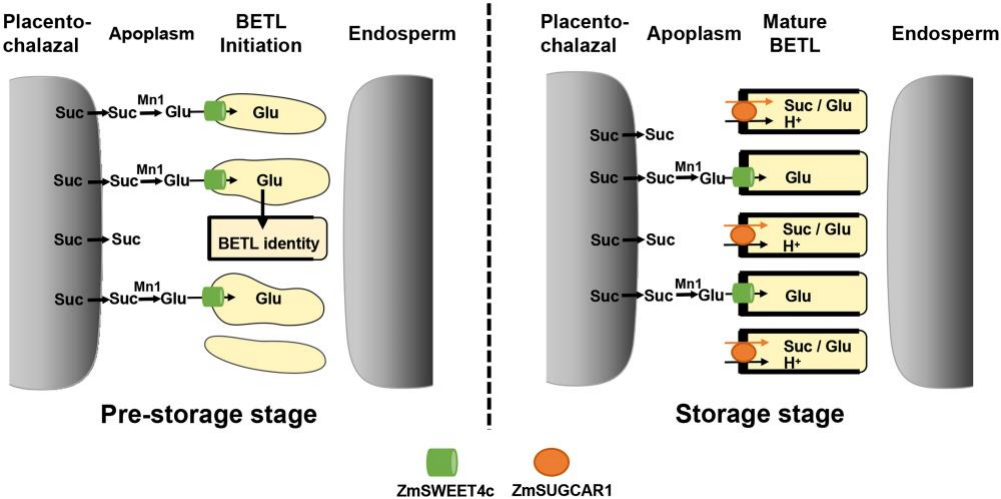
A working model of ZmSUGCAR1 in regulating grain filling in maize. ZmSUGCAR1 acts as an H+ and sucrose (Suc)/glucose (Glu) symporter to transfer sugars from the maternal placenta–chalazal region into the endosperm across the mature BETL (storage stage), circumventing sucrose hydrolysis. Meanwhile, SWEET4c also operates for glucose transport after BETL establishment (prestorage stage). Reprinted from Yang et al. (2022) Figure 9C.

The yield, quality, and nutritional value of seeds are complex traits whose development depends on the transport of sugars and other nutrients from maternal to filial tissues. The identification of nutrient transporters and their mechanisms of action in seeds can lay a foundation for breeding varieties with improved grain yield and quality. Last year, **Bo Yang and colleagues** (Yang et al. 2022) identified an unexpected sugar transporter in maize. While screening an ethyl methanesulfonate–mutagenized library for homozygous mutants of maize, the authors identified a line with altered kernel size named *poorly filled kernel2109* (*pfk2109*). Using map-based cloning and sequencing, they identified the mutant gene, previously annotated as a nitrate transporter 1/peptide transporter (NRT1/PTR). NRT1/PTR-type transporters are known to transport nitrate, peptides, and ions (Léran et al. 2014). Due to the defects in sugar loading during the mutant grain development, Yang and coauthors (2022) renamed this maize gene as Sucrose and Glucose Carrier 1 (ZmSUGCAR1). The authors found that the ZmSUGCAR1 mRNA expression peak coincides with the most active grain-filling (storage) stage, and the proteins were specifically expressed in the basal endosperm transfer layer (BETL), which mediates nutrient transfer from maternal to seed tissue. The authors then generated complementation and CRISPR-Cas9–mediated knockout lines and showed that ZmSUGCAR1 acts as a sugar transporter able to import sucrose and glucose into the seed endosperm. Furthermore, the authors showed that ZmSUGCAR1 contributes to potassium loading but does not affect nitrate accumulation in the kernels. This study identified an unexpected sugar transporter crucial for maize grain development that complements the actions of other well-known sugar transporters, such as SWEETs (Fig. 1).

## November 2018: Innovation by whole-genome duplication

Gene duplication is commonly observed in plant genomes and often leads to evolutionary novelty such as new gene functions. About 75% of flowering plants originated after consecutive whole-genome duplications commonly referred to as the gamma (γ) triplication event (Jiao et al. 2012). **Zhicheng Zhang and coauthors** (Zhang et al. 2018) decided to investigate the effect of the γ event on the MADS-box transcription factors. They used statistical methods to reconstruct protein interaction networks (PINs) between representatives of different MADS-box gene subfamilies before and after the γ triplication and from the origin of Arabidopsis and tomato. They reconstructed 26 ancestral proteins with high confidence, and by synthesizing their DNA sequences and performing a high-throughput yeast 2-hybrid (Y2H) assay, they genetically validated their protein-protein interactions. Zhang et al. (2018) also looked at the gene dosages of the reconstructed ancestral proteins that interacted before the γ event by comparing their Y2H data with other MADS PINs described in the literature. Overall, the interaction patterns of ancestral proteins were more similar to those of Arabidopsis-PIN and tomato-PIN than the interactions of proteins after the γ event. This indicated that the γ event led to the expansion and retention of MADS proteins more strongly than subsequent genomic events. The authors performed a series of network analyses and found that despite the γ triplication, a rapid rewiring of the PIN took place and a number of specific interactions were maintained during MADS evolution; therefore, the topology of the network was not qualitatively affected. They also observed that often innovation of the MADS-PIN would occur by neoredundancy, where 2 proteins interact with a third whereas the ancestor did not. The study of Zhang et al. (2018) sheds light on the origin and evolutionary diversification of MADS protein interactions and provides a rich resource to understand the effect of the γ triplication event on plant evolution.

## November 1998: Cooperative DNA binding by Opaque2

Opaque2 (O2) is a basic leucine zipper transcriptional activator first described as modulating the expression of several seed-storage protein (SSP) genes during seed development (Schmidt et al. 1992). In 1998, **José A. Yunes and coauthors** (Yunes et al. 1998) investigated the mechanism of O2 binding in the promoter of a target gene involved with the synthesis of SSPs in Coix (*Coix lacryma-jobi* L.) called α-coixin. The authors generated mutated versions of the *O2* homologs in Coix and different sizes of the target binding sequence of the α-coixin gene; using electrophoretic mobility shift assays, they found that there are 2 adjacent binding sites in the α-coixin promoter at which 2 O2 homodimers can simultaneously bind. They then generated a series of mutants for the 2 adjacent binding sites and used quantitative Dnase I footprint assays and transient expression assays in tobacco protoplasts to determine whether O2 binding requires both sites to be closely located and the biological consequences of this. They found that all mutations decreased the binding efficiency of the Coix O2 protein, but mutation of 1 or 2 nucleotides at 1 site did not affect the O2 binding to the adjacent site. Furthermore, they showed that O2 proteins cooperatively binds to the adjacent target sites. The work of Yunes et al. (1998) describes the mechanism of action of O2 and sheds light on the control of SSP production and accumulation during seed development.

## Dive Curated Terms

The following phenotypic, genotypic, and functional terms are of significance to the work described in this paper:
